# Supplementary material for: Resolving the full spectrum of human genome variation using Linked-Reads
Source: Genome Res. 2019 Apr;29(4):635–45. doi: 10.1101/gr.234443.118 (PMC6442396; doi:10.1101/gr.234443.118)
Supplement: Supplemental Material [file supp_gr.234443.118_Supplemental_longranger-2.2.zip › longranger-2.2.tar.bz2!/lr2.2-public/lib/stan/cmdstan/stan/doc/api/html/classstan_1_1json_1_1json__data.html]

Stan: stan::json::json\_data Class Reference


|  |  |
| --- | --- |
|  | Stan  2.5.0  probability, sampling & optimization |


- Main Page
- Related Pages
- Namespaces
- Classes
- Files

- Class List
- Class Index
- Class Hierarchy
- Class Members

All Classes Namespaces Files Functions Variables Typedefs Enumerations Enumerator Friends Macros Pages

- stan
- json
- json\_data

Public Member Functions |
List of all members

stan::json::json\_data Class Reference

A `json_data` is a `var_context` object that represents a set of named values which are typed to either `double` or `int` and can be either scalar value or a non-empty array of values of any dimensionality.
More...

`#include <json_data.hpp>`

Inheritance diagram for stan::json::json\_data:

|  |  |
| --- | --- |
| Public Member Functions | |
|  | json\_data (std::istream &in) |
|  | Construct a json\_data object from the specified input stream. More... |
|  | |
| bool | contains\_r (const std::string &name) const |
|  | Return `true` if this json\_data contains the specified variable name. More... |
|  | |
| bool | contains\_i (const std::string &name) const |
|  | Return `true` if this json\_data contains an integer valued array with the specified name. More... |
|  | |
| std::vector< double > | vals\_r (const std::string &name) const |
|  | Return the double values for the variable with the specified name or null. More... |
|  | |
| std::vector< size\_t > | dims\_r (const std::string &name) const |
|  | Return the dimensions for the variable with the specified name. More... |
|  | |
| std::vector< int > | vals\_i (const std::string &name) const |
|  | Return the integer values for the variable with the specified name. More... |
|  | |
| std::vector< size\_t > | dims\_i (const std::string &name) const |
|  | Return the dimensions for the integer variable with the specified name. More... |
|  | |
| virtual void | names\_r (std::vector< std::string > &names) const |
|  | Return a list of the names of the floating point variables in the json\_data. More... |
|  | |
| virtual void | names\_i (std::vector< std::string > &names) const |
|  | Return a list of the names of the integer variables in the json\_data. More... |
|  | |
| bool | remove (const std::string &name) |
|  | Remove variable from the object. More... |
|  | |
| Public Member Functions inherited from stan::io::var\_context | |
| virtual | ~var\_context () |
|  | |
| void | add\_vec (std::stringstream &msg, const std::vector< size\_t > &dims) const |
|  | |
| void | validate\_dims (const std::string &stage, const std::string &name, const std::string &base\_type, const std::vector< size\_t > &dims\_declared) const |
|  | |

|  |  |
| --- | --- |
| Additional Inherited Members | |
| Static Public Member Functions inherited from stan::io::var\_context | |
| static std::vector< size\_t > | to\_vec () |
|  | |
| static std::vector< size\_t > | to\_vec (size\_t n1) |
|  | |
| static std::vector< size\_t > | to\_vec (size\_t n1, size\_t n2) |
|  | |
| static std::vector< size\_t > | to\_vec (size\_t n1, size\_t n2, size\_t n3) |
|  | |
| static std::vector< size\_t > | to\_vec (size\_t n1, size\_t n2, size\_t n3, size\_t n4) |
|  | |
| static std::vector< size\_t > | to\_vec (size\_t n1, size\_t n2, size\_t n3, size\_t n4, size\_t n5) |
|  | |
| static std::vector< size\_t > | to\_vec (size\_t n1, size\_t n2, size\_t n3, size\_t n4, size\_t n5, size\_t n6) |
|  | |
| static std::vector< size\_t > | to\_vec (size\_t n1, size\_t n2, size\_t n3, size\_t n4, size\_t n5, size\_t n6, size\_t n7) |
|  | |
| static std::vector< size\_t > | to\_vec (size\_t n1, size\_t n2, size\_t n3, size\_t n4, size\_t n5, size\_t n6, size\_t n7, size\_t n8) |
|  | |

## Detailed Description

A `json_data` is a `var_context` object that represents a set of named values which are typed to either `double` or `int` and can be either scalar value or a non-empty array of values of any dimensionality.

Arrays must be retangular and the values of an array are all of the same type, either double or int.

The dimensions and values of variables are accessed by variable name. The values of a variable are stored as a vector of values and a vector of array dimensions, where a scalar value consists of a single value and an emtpy vector for the dimensionality. Multidimensional arrays are stored in column-major order, meaning the first index changes the most quickly. If all the values of an array are int values, the array will be stored as a vector of ints, else the array will be stored as a vector of type double.

`json_data` objects are created by using the `json_parser` and a `json_data_handler` to read a single JSON text from an input stream.

Definition at line 46 of file json\_data.hpp.

## Constructor & Destructor Documentation

|  |  |  |  |  |  |
| --- | --- | --- | --- | --- | --- |
| stan::json::json\_data::json\_data | ( | std::istream & | *in* | ) |  |

inline

Construct a json\_data object from the specified input stream.

**Warning:** This method does not close the input stream.

Parameters
:   |  |  |
    | --- | --- |
    | in | Input stream from which to read. |

Exceptions
:   |  |  |
    | --- | --- |
    | json\_exception | if data is not well-formed stan data declaration |

Definition at line 78 of file json\_data.hpp.

## Member Function Documentation

|  |  |  |  |  |  |
| --- | --- | --- | --- | --- | --- |
| bool stan::json::json\_data::contains\_i | ( | const std::string & | *name* | ) | const |

inlinevirtual

Return `true` if this json\_data contains an integer valued array with the specified name.

Parameters
:   |  |  |
    | --- | --- |
    | name | Variable name to test. |

Returns
:   `true` if the variable name has an integer array value.

Implements stan::io::var\_context.

Definition at line 103 of file json\_data.hpp.

|  |  |  |  |  |  |
| --- | --- | --- | --- | --- | --- |
| bool stan::json::json\_data::contains\_r | ( | const std::string & | *name* | ) | const |

inlinevirtual

Return `true` if this json\_data contains the specified variable name.

This method returns `true` even if the values are all integers.

Parameters
:   |  |  |
    | --- | --- |
    | name | Variable name to test. |

Returns
:   `true` if the variable exists.

Implements stan::io::var\_context.

Definition at line 91 of file json\_data.hpp.

|  |  |  |  |  |  |
| --- | --- | --- | --- | --- | --- |
| std::vector<size\_t> stan::json::json\_data::dims\_i | ( | const std::string & | *name* | ) | const |

inlinevirtual

Return the dimensions for the integer variable with the specified name.

Parameters
:   |  |  |
    | --- | --- |
    | name | Name of variable. |

Returns
:   Dimensions of variable.

Implements stan::io::var\_context.

Definition at line 165 of file json\_data.hpp.

|  |  |  |  |  |  |
| --- | --- | --- | --- | --- | --- |
| std::vector<size\_t> stan::json::json\_data::dims\_r | ( | const std::string & | *name* | ) | const |

inlinevirtual

Return the dimensions for the variable with the specified name.

Parameters
:   |  |  |
    | --- | --- |
    | name | Name of variable. |

Returns
:   Dimensions of variable.

Implements stan::io::var\_context.

Definition at line 135 of file json\_data.hpp.

|  |  |  |  |  |  |
| --- | --- | --- | --- | --- | --- |
| virtual void stan::json::json\_data::names\_i | ( | std::vector< std::string > & | *names* | ) | const |

inlinevirtual

Return a list of the names of the integer variables in the json\_data.

Parameters
:   |  |  |
    | --- | --- |
    | names | Vector to store the list of names in. |

Implements stan::io::var\_context.

Definition at line 191 of file json\_data.hpp.

|  |  |  |  |  |  |
| --- | --- | --- | --- | --- | --- |
| virtual void stan::json::json\_data::names\_r | ( | std::vector< std::string > & | *names* | ) | const |

inlinevirtual

Return a list of the names of the floating point variables in the json\_data.

Parameters
:   |  |  |
    | --- | --- |
    | names | Vector to store the list of names in. |

Implements stan::io::var\_context.

Definition at line 178 of file json\_data.hpp.

|  |  |  |  |  |  |
| --- | --- | --- | --- | --- | --- |
| bool stan::json::json\_data::remove | ( | const std::string & | *name* | ) |  |

inline

Remove variable from the object.

Parameters
:   |  |  |
    | --- | --- |
    | name | Name of the variable to remove. |

Returns
:   If variable is removed returns `true`, else returns `false`.

Definition at line 205 of file json\_data.hpp.

|  |  |  |  |  |  |
| --- | --- | --- | --- | --- | --- |
| std::vector<int> stan::json::json\_data::vals\_i | ( | const std::string & | *name* | ) | const |

inlinevirtual

Return the integer values for the variable with the specified name.

Parameters
:   |  |  |
    | --- | --- |
    | name | Name of variable. |

Returns
:   Values.

Implements stan::io::var\_context.

Definition at line 151 of file json\_data.hpp.

|  |  |  |  |  |  |
| --- | --- | --- | --- | --- | --- |
| std::vector<double> stan::json::json\_data::vals\_r | ( | const std::string & | *name* | ) | const |

inlinevirtual

Return the double values for the variable with the specified name or null.

Parameters
:   |  |  |
    | --- | --- |
    | name | Name of variable. |

Returns
:   Values of variable.

Implements stan::io::var\_context.

Definition at line 114 of file json\_data.hpp.

---

The documentation for this class was generated from the following file:

- src/stan/io/json/json\_data.hpp


---

[ Stan Home Page ]

*© 2011–2014,
Stan Development Team.*
